# Transfer learning of condition-specific perturbation in gene interactions improves drug response prediction

**DOI:** 10.1093/bioinformatics/btae249

**Published:** 2024-06-28

**Authors:** Dongmin Bang, Bonil Koo, Sun Kim

**Affiliations:** Interdisciplinary Program in Bioinformatics, Seoul National University, Seoul, 08826, Republic of Korea; AIGENDRUG Co., Ltd., Seoul, 08758, Republic of Korea; Interdisciplinary Program in Bioinformatics, Seoul National University, Seoul, 08826, Republic of Korea; AIGENDRUG Co., Ltd., Seoul, 08758, Republic of Korea; Interdisciplinary Program in Bioinformatics, Seoul National University, Seoul, 08826, Republic of Korea; AIGENDRUG Co., Ltd., Seoul, 08758, Republic of Korea; Department of Computer Science and Engineering, Seoul National University, Seoul, 08826, Republic of Korea; Interdisciplinary Program in Artificial Intelligence, Seoul National University, Seoul, 08826, Republic of Korea

## Abstract

**Summary:**

Drug response is conventionally measured at the cell level, often quantified by metrics like IC50. However, to gain a deeper understanding of drug response, cellular outcomes need to be understood in terms of pathway perturbation. This perspective leads us to recognize a challenge posed by the gap between two widely used large-scale databases, LINCS L1000 and GDSC, measuring drug response at different levels—L1000 captures information at the gene expression level, while GDSC operates at the cell line level. Our study aims to bridge this gap by integrating the two databases through transfer learning, focusing on condition-specific perturbations in gene interactions from L1000 to interpret drug response integrating both gene and cell levels in GDSC. This transfer learning strategy involves pretraining on the transcriptomic-level L1000 dataset, with parameter-frozen fine-tuning to cell line-level drug response. Our novel condition-specific gene–gene attention (CSG^2^A) mechanism dynamically learns gene interactions specific to input conditions, guided by both data and biological network priors. The CSG^2^A network, equipped with transfer learning strategy, achieves state-of-the-art performance in cell line-level drug response prediction. In two case studies, well-known mechanisms of drugs are well represented in both the learned gene–gene attention and the predicted transcriptomic profiles. This alignment supports the modeling power in terms of interpretability and biological relevance. Furthermore, our model’s unique capacity to capture drug response in terms of both pathway perturbation and cell viability extends predictions to the patient level using TCGA data, demonstrating its expressive power obtained from both gene and cell levels.

**Availability and implementation:**

The source code for the CSG^2^A network is available at https://github.com/eugenebang/CSG2A.

## 1 Introduction

Drug response prediction plays a pivotal role in cancer treatment and personalized medicine. Drug response can be defined at multiple levels, including the gene (transcriptome) level, cell line (*in vitro*) level, and patient (clinical) level. At the transcriptomic-level, the task involves predicting perturbed gene expression profiles in response to chemical treatments (Pham *et al.* 2021, Zhu *et al.* 2021). Cell line-level drug response prediction utilizes basal gene expression profiles and chemical information to predict cell viability measures like inhibitory concentration 50 (IC50) values (Garnett *et al.* 2012, Rees *et al.* 2016), while patient-level prediction aims to distinguish responders from non-responders (Ding *et al.* 2016, Huang *et al.* 2020).

Conventionally, drug response has been measured at the cell level, often quantified by metrics such as IC50. Utilizing large-scale databases, predicting cell line-level drug responses from transcriptomic profiles is well-explored (Chen and Zhang 2021, Partin *et al.* 2023), supported by biological relevance as drug–target binding initiates pathway perturbations influencing cellular outcomes (Pak *et al.* 2023). This cascade of perturbation propagates through the intracellular gene–gene network, subsequently influencing changes in both transcriptomic and cellular measurements. Hence, a deeper understanding of drug response requires modeling intricate pathway perturbations influencing cellular outcomes.

However, this comprehensive endeavor faces a substantial challenge arising from the existing gap between two extensively utilized drug response databases: LINCS L1000 (Library of Integrated Network-based Cellular Signatures) (Subramanian *et al.* 2017) and GDSC (Genomics of Drug Sensitivity in Cancer) (Garnett *et al.* 2012). LINCS provides gene-level drug response measures, while GDSC operates at the cell line level. This measurement level gap presents a substantial obstacle in comprehensive int egration for modeling drug response mechanisms.

On the other hand, existing models focus on single-level drug responses, particularly at the cell level, overlooking the essential integration of gene-level information for comprehensive modeling of drug response. AutoEncoder-based approaches (Chiu *et al.* 2019, Rampášek *et al.* 2019) project omics profiles into lower dimensions, losing gene context and interactions. Graph neural network (GNN)-based approaches (Nguyen *et al.* 2022, Shin *et al.* 2022) face additional limitations due to non-condition-specific knowledge, such as fixed structures protein–protein interaction networks or pathway information.

**Figure 1.**
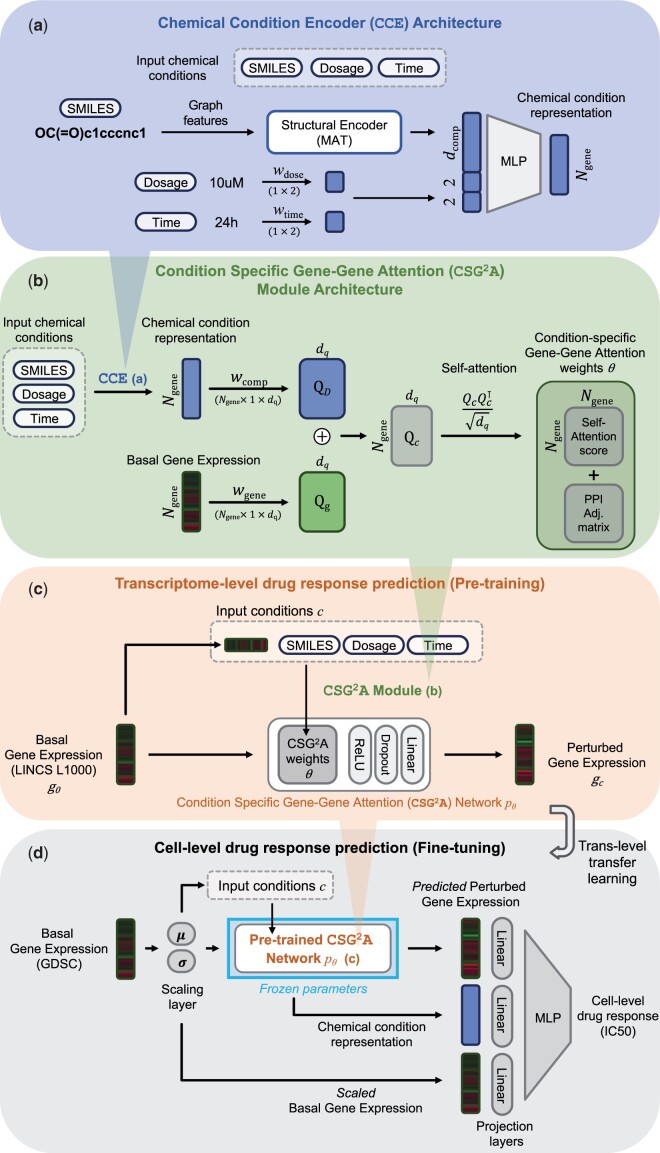
Overview and architecture of proposed framework. (a) Chemical condition encoder (CCE): built upon the pretrained structural encoder MAT, CCE generates chemical condition representations from SMILES, dosage, and treatment duration. (b) Condition-specific gene–gene attention (CSG^2^A) module: taking chemical conditions and basal gene expression as input, CSG^2^A module conducts self-attention after combining compound and gene expression profile representations. The resulting attention scores are summed with the PPI adjacency matrix, producing the weights θ for the CSG^2^A network. (c) Pretraining on transcriptome level drug response prediction (LINCS L1000): the basal gene expression profile undergoes CSG^2^A network $p_{\theta }$for predicting chemical-induced perturbed gene expression. Computational details of CSG^2^A from input conditions, basal gene expression, and chemical conditions are shown in panel b. (d) Fine-tuning on Cell line-level drug response (GDSC dataset): For trans-level transfer learning, a trainable scaling layer is introduced before the pretrained CSG^2^A network. Using the predicted perturbed gene expression and chemical condition representation from the pretrained CSG^2^A network, joined with scaled basal gene expression, the prediction head layers outputs the IC50 value.

In response to these challenges, we introduce a novel approach that is to bridge the gap between LINCS L1000 and GDSC. We revisit attention mechanisms to model chemical-induced gene–gene network perturbations and employ a transfer learning approach to learn from the transcriptomic landscape, transferring knowledge obtained from gene-level to cell line-level drug responses (Fig. 1).

Our trans-level transfer learning involves pretraining on LINCS L1000 and fine-tuning on cell line-level drug responses. This procedure enables our model to adeptly capture the chemical-induced perturbations in gene interactions, comprehensively understanding gene-level interactions for transfer to higher-level drug response. The key component of our approach is the condition-specific gene–gene attention (CSG^2^A) mechanism, designed to dynamically learn gene interactions specific to input conditions, guided by both data and biological network priors.

Since its re-discovery by the transformer model (Vaswani *et al.* 2017), attention mechanism plays a crucial role in efficiently learning relevant relations of input entities through all-pairwise similarity computation. Harnessing the expressiveness of self-attention, our CSG^2^A mechanism efficiently learns dependencies among genes at the transcriptome scale without relying on predefined features. Additionally, our unique design choice of leveraging attention scores as neural network parameters facilitates seamless knowledge transfer. To further guide this learning process with prior knowledge, we introduce a protein–protein interaction (PPI) adjacency matrix.

Our CSG^2^A network, coupled with trans-level transfer learning strategy, achieves state-of-the-art performance compared to existing drug response prediction models on the GDSC dataset. Case studies further validate the alignment of learned CSG^2^A with the known mode of action of drugs, highlighting the interpretability and biological relevance of our model. Additional experiments demonstrate its adaptability to predict drug responses in cancer patients from the TCGA dataset.

## 2 Related work

### 2.1 Large-scale drug response datasets

#### 2.1.1 Gene (transcriptome) level

The LINCS connectivity map (CMAP) L1000 (Subramanian *et al.* 2017) is a large-scale pharmacogenomics screening dataset from the LINCS. This dataset contains flow cytometry-based transcriptomic responses of cell lines to various perturbations, covering a wide spectrum of chemical treatments, cell types, and experimental conditions. The landmark genes in the LINCS L1000 dataset refer to a selected representative 978 genes directly measured across all assays, providing a condensed snapshot of cellular transcriptomic dynamics.

#### 2.1.2 Cell line (*in vitro*) level

There are several comprehensive resources for understanding the relationship between genomic features and drugs at the cell line level: GDSC (Garnett *et al.* 2012) and NCI-60 (Shoemaker 2006). GDSC, one of the representative ones, provides drug sensitivity profiles for a variety of anti-cancer drugs, illustrating how different cancer cell lines respond to these drugs based on their genomic features.

#### 2.1.3 Patient (clinical) level

The Cancer Genome Atlas (TCGA) (Weinstein *et al.* 2013) is a crucial resource that provides molecular characterization data for patients with various types of cancer. While TCGA primarily focuses on understanding the genomic landscape of cancers, it helps identify therapeutic targets, biomarkers, and patient subgroups, ultimately contributing to the development of more effective and personalized cancer treatments.

### 2.2 Drug response prediction in multiple levels

There has been various deep-learning approaches proposed for predicting drug responses on the cell level including autoencoders (Chiu *et al.* 2019, Rampášek *et al.* 2019), GNNs (Nguyen *et al.* 2022, Shin *et al.* 2022), and other architectures (Deng *et al.* 2020, Chawla *et al.* 2022, Jiang *et al.* 2022) (Supplementary Methods). However, multi-leveled transfer learning strategy from the gene level to higher levels has been rarely explored.

Dr.VAE (Rampášek *et al.* 2019) is the only work attempting to transfer knowledge from gene expression level drug response to cell line drug response prediction. Utilizing the Variational AutoEncoder framework, the authors pretrained models on LINCS L1000 data and applied additional classifiers to predict cell line-level drug responses. However, Dr.VAE trains separate models for each drug without consideration of various chemical treatment conditions, only allowing transfer learning on the drugs enlisted in the LINCS L1000 dataset. This model design resulted in a limited ability to predict responses across various drugs and treatment conditions effectively.

## 3 Materials and methods

### 3.1 Condition-specific gene–gene attention network

We define the problem of predicting chemical-perturbed gene expression profile, denoted as $g_{c}$, as the design of a condition-specific prediction model $p_{\theta }$. The predictive framework for the chemical-induced transcriptomic profile with the condition-specific neural network $p_{\theta }$ with parameters θ can be expressed as:

$$\begin{array}{c} g_{c}=p_{\left(\theta |c\right)}(g_{0})=p_{\left(\theta |g_{0},S,d,t\right)}(g_{0}). \end{array}$$

An essential aspect of this formulation is the explicit dependence of the parameters θ on the set of conditions $c=(g_{0},S,d,t)$. Here, $g_{0}$ represents the basal gene expression profile before compound treatment, *S* denotes the chemical structure, and *d* and *t* as treatment dose and time, respectively. This formulation establishes a comprehensive framework for the prediction of chemical-induced gene expression profiles, where the neural network’s behavior is intricately linked to the specific conditions.

#### 3.1.1 Chemical condition encoder

Chemical-induced transcriptomic perturbation results from a biological cascade initiated by the binding of a chemical to its target proteins. The impact of this perturbation is mainly dictated by the chemical’s structure, with additional dependencies on exposure dosage and time. Therefore, encoding the chemical condition necessitates incorporating not only structural information but also dosage and time factors.

To model the chemical condition comprehensively, we introduce a chemical condition encoder (CCE) (Fig. 1a). This encoder takes structural features *S*, dosage *d*, and time *t* as input for producing a chemical condition representation $D_{S,d,t}$.

The base component of our chemical structure encoder is the pretrained molecular attention transformer (MAT) (Maziarka *et al.* 2020). MAT is a transformer-based structure encoder pretrained on a masked entity prediction task with two million compounds from the ZINC15 database (Sterling and Irwin 2015). We leverage the pretrained weights provided by the authors throughout our experiments. The dosage and time conditions, scaled by 100μM and 72 h, respectively, are expanded to two dimensions by corresponding linear layers. The concatenated vector of these two expanded vectors and the MAT representation is then passed on to the final multi-layer perceptron (MLP) layer.

The mathematical representation of the chemical condition $D_{S,d,t}\in R^{N_{\text{gene}}}$ can be expressed as:

$$D_{S,d,t}={\text{MLP}}_{\mathrm{CCE}}([\mathrm{MAT}(S),d\cdot W_{\text{dose}},t\cdot W_{\text{time}}]).$$

Here, [·,·] refers to the concatenation operation, $W_{\text{dose}},W_{\text{time}}\in R^{1\times 2}$ are linear weights for dosage and time encoding, respectively, and MLP${\;}_{\mathrm{CCE}}$ denotes the final MLP layer.

The overall process generates the final chemical condition representation aligned with the dimension of the number of genes ($N_{\text{gene}}$). This representation is further utilized by the CSG^2^A module for modeling the perturbed gene-gene network.

#### 3.1.2 CSG^2^A network

To predict the chemical-induced gene expression profile, we incorporate CSG^2^A values as our neural network parameters θ (Fig. 1b). This network is designed to capture the intricate relationships between genes in the treatment condition *c* under the influence of basal gene expression $g_{0}$, chemical structure *S*, dosage *d* and time *t*.

The process begins with the basal gene expression profile $g_{0}$ and chemical representation $D_{S,d,t}$ obtained from the CCE. Then, the CSG^2^A module introduces a novel paradigm by treating neural network parameters as CSG^2^A values. This differentiation from conventional neural networks, where parameters are randomly initialized, enables the model to learn the conditional effects of both gene expression and drugs.

The calculation of attention values involves self-attention on the gene-level condition representation $Q_{\left(C|g_{0},S,d,t\right)}\in R^{N_{\text{gene}}\times h}$. This self-attention module allows for the explicit learning of attention scores in a gene-specific manner, which is crucial for capturing gene expression perturbations effectively. The gene-level condition representation is defined as the sum of the basal gene expression representation and the chemical condition representation:

$$\begin{array}{c} Q_{\left(C|g_{0},S,d,t\right)}=Q_{g}+Q_{D},\\ Q_{g}=W_{\text{gene}}\cdot g_{0},\;Q_{D}=W_{\text{comp}}\cdot D_{S,d,t}, \end{array}$$

where $W_{\text{gene}}$ and $W_{\text{comp}}\in R^{N_{\text{gene}}\times 1\times h}$ represent learnable linear weights for the dimension expansion of gene expression and chemical condition representations into hidden dimension *h*. The utilization of a three-dimensional weight tensor $W\in R^{N_{\text{gene}}\times 1\times h}$ with distinct weights for each gene ensures that both the input expression value and the context of each gene are considered during dimension expansion.

Subsequently, the dot product attention score matrix $A_{Q_{C}}$ is calculated as $\alpha_{i,j}=Q_{Ci}\cdot Q_{Cj}^{⊺}$, where $\alpha_{i,j}$ denotes the attention score between genes *i* and *j*. To enhance ability to capture higher-order gene interactions in a biological context and reduce the search space by providing a starting point of the interactions and the scales, we integrate a PPI adjacency matrix ($A_{\text{PPI}}$) as prior knowledge as: $A_{\mathrm{CSG2A}}=A_{Q_{C}}+A_{\text{PPI}}$.

The calculated attention score matrix directly serves as neural network weights. The first layer of the CSG^2^A network is a linear layer with weights equal to $A_{\mathrm{CSG2A}}$. The network further includes an activation layer, a dropout layer, and a final linear layer. This comprehensive design allows the CSG^2^A network to effectively capture and leverage condition-specific gene–gene interactions for precise predictions of perturbed gene expression. The detailed hyperparameters and their search space are detailed in Supplementary Table S1.

### 3.2 Trans-level transfer learning strategy

In order to learn the chemical-perturbed gene network from the transcriptome data and leverage such model for higher-level drug response predictions, we implement a trans-level transfer learning strategy.

#### 3.2.1 LINCS pretraining

In the LINCS pretraining phase (Fig. 1c), our model focuses on predicting the chemical-induced transcriptome profile, a crucial step in capturing the intricate dynamics of gene expression alterations under various chemical conditions. The inputs to this phase consist of the basal gene expression profile and the corresponding chemical structure for predicting the perturbed gene expression. Notably, our model also takes into account additional factors such as drug dosage and treatment time, enhancing its capacity to comprehend the nuanced aspects of chemical-induced transcriptomic changes. During training, our model utilizes mean-squared error (MSE) loss on the perturbed gene expression profile, aiming to minimize the gap between predicted and actual values.

A key highlight of the LINCS pretraining phase is the training of the CSG^2^A network. This network plays a pivotal role in learning condition-specific gene–gene interactions, contributing to the accurate prediction of perturbed gene expression profiles. By leveraging the diverse and extensive gene-level data available in LINCS, our model adapts to variations in gene expression induced by different chemical conditions. This acquired knowledge plays a critical role in the subsequent fine-tuning process, ensuring the model’s adaptability and effectiveness in predicting in vitro drug responses during the later stages of our trans-level transfer learning strategy.

#### 3.2.2 Fine-tuning on GDSC

In the GDSC fine-tuning phase (Fig. 1d), our model undergoes further refinement to seamlessly adapt to the *in vitro* drug response dynamics observed in the GDSC dataset. The inputs for the fine-tuning task are consistent as the pretraining stage, including the basal gene expression profile and the corresponding chemical condition. However, the target value shifts to predicting the log IC50 value, a key metric in quantifying the drug sensitivity of cancer cells.

To facilitate a smooth trans-level transfer, accounting for potential batch effects across different datasets, a trainable scaling layer is strategically introduced. This layer serves as a crucial bridge between the pretrained CSG^2^A network and the GDSC dataset. Notably, we intentionally eliminate all bias terms within the layers of our neural network, enhancing the adaptability of the transfer process between distinct transcriptomic spaces.

The introduced scalable layer incorporates two learnable latent variables: mean (μ) and standard deviation (σ). By passing through the scaling equation $g_{0}^{\prime }=(g_{0}-\mu )/\sigma$, the input basal gene expression profile ($g_{0}$) is aligned with the pretrained LINCS space. It is important to note that both the mean and standard deviation values function as latent variables, not having a preassigned target values. This design choice allows the model to autonomously learn and adapt its scaling parameters, contributing to its transferability to higher-level drug response tasks. The training target is the log IC50 value, and the model is optimized to minimize the disparity between predicted log IC50 values and actual values using the MSE loss.

An empirical observation emphasizes the critical role of the LINCS pretraining phase in learning gene–gene attention, achieving optimal prediction performance with fully frozen pretrained parameters. Further details and insights into this observation are discussed in the Results section.

Lastly, it is important to note that the cell viability data from GDSC does not include information on the specific drug-treated conditions, such as dosage and treatment time. Therefore, to maintain consistency and align with the GDSC dataset’s data generation process, we utilized the widely accepted treatment time of 72 h for IC50 measurement and the dosage at 10 µM, commonly used in LINCS L1000 dataset and also cell viability measurements, for all experiments.

### 3.3 Dataset and metrics

#### 3.3.1 LINCS L1000 dataset

We downloaded the LINCS phase 1 data from GEO with accession number GSE92742 (Subramanian *et al.* 2017). Since DMSO is used as a control corresponding to the compound, transcriptome data were obtained from the sample treated with DMSO as basal gene expression. A total of 649 batches containing samples treated with DMSO were obtained, and from these batches, 202 962 transcriptomic profile of samples treated with compounds were obtained. We utilized the 978 landmark genes that were measured when the LINCS data was produced.

#### 3.3.2 GDSC dataset

We obtained basal gene expression profile for cell lines from cell model passports (Garcia-Alonso *et al.* 2018). The ${\;\text{log}\;}_{2}$FPKM values were transformed into robust *z*-scores to be used as input at the same level as the LINCS data. The robust *z*-score is computed using the following equation:

$$z_{i}=\frac{x_{i}-\text{median}(X)}{1.4826\cdot \text{MAD}(X)},$$

where MAD indicates the median absolute deviation, *X* represents the expression values for a gene for all samples in the data, $x_{i}$ is the expression level of a sample *i*, and $z_{i}$ is a robust z-score for the gene of sample *i*. Additionally, the drug response values (i.e. log IC50) for each cell line were obtained from GDSC (Garnett *et al.* 2012).

#### 3.3.3 TCGA dataset

Using the TCGA classification information of cell lines provided by GDSC, transcriptome data of patients corresponding to tumor samples for 21 cancer types were obtained from UCSC Xena (Goldman *et al.* 2020). For each cancer type, GDSC data and TCGA data were batch-corrected using Combat (Johnson *et al.* 2007) at the ${\;\text{log}\;}_{2}$FPKM level and then converted to a robust *z*-score. Curated data on drug treatment and responsiveness in TCGA patients were obtained from the supplementary data provided in Ding *et al.* (2016).

#### 3.3.4 PPI network

STRING v12.0 (Szklarczyk *et al.* 2023) was employed as the biological network for prior knowledge to guide the gene-gene interaction learning process. To ensure the inclusion of confident edges, edges with a combined score greater than 900 were selected.

## 4 Results and discussion

### 4.1 Performance on cell line drug response prediction

First, we applied our framework and compared with other state-of-the-art models in predicting cell line drug responses of the GDSC dataset. Aligning with the comprehensive investigation by Partin *et al.* (2023), our evaluation encompassed a 10-fold cross-validation, employing four distinct data partitioning schemes further detailed in Supplementary Methods section.

Among the eight comparison models in our evaluation, two were machine learning algorithms—random forest (RF) and support vector machines (SVM). These models utilized Morgan molecular fingerprints and gene expression values as input features. Additionally, six deep-learning methods (GraphDRP, PathDNN, Precily, DRPreter, DeepCoVDR, and DeepTTA) were included in the comparative analysis.

Performance metrics were assessed in terms of root mean square error (RMSE, Equation (1)) and Pearson correlation coefficient (PCC, Equation (2)), measuring the distance and correlation between predicted and true log IC50 values, respectively

(1) $$\text{RMSE}=\sqrt{\frac{1}{n}\sum_{i=1}^{n}{\left(y_{i}-{\hat{y}}_{i}\right)}^{2}}.$$

 

(2) $$\text{PCC}=\frac{\sum_{i=1}^{n}\left(y_{i}-\bar{y}\right)({\hat{y}}_{i}-\bar{\hat{y}})}{\sqrt{\sum_{i=1}^{n}{\left(y_{i}-\bar{y}\right)}^{2}}\sqrt{\sum_{i=1}^{n}{\left({\hat{y}}_{i}-\bar{\hat{y}}\right)}^{2}}}.$$

Here, *n* represents the number of samples, $y_{i}$ is the true value and ${\hat{y}}_{i}$ is the predicted value of log IC50 for sample *i*. Also, $\bar{y}$ and $\bar{\hat{y}}$ are the means of each values.

Remarkably, our proposed model demonstrated state-of-the-art performance across all four partitioning schemes, exhibiting significant improvements, particularly in challenging drug-blind and disjoint-set scenarios (Table 1). While existing deep-learning approaches have shown improved performances in mixed-set settings, they often exhibit a lack of generalizability, leading to noticeable decreases in performance in harsh split settings. This phenomenon may stem from the widely-observed overfitting tendency of deep-learning models, allowing conventional machine learning algorithms to outperform them. However, our CSG^2^A network consistently achieved the best performances in all splits, indicating the robust generalizability of our condition-specific pretraining approach to challenging validation settings.

**Table 1.** Cell line drug response prediction performances on GDSC dataset with four different partitioning schemes.

	Mixed-set		Cell line-blind		Drug-blind		Disjoint-set	
Models	RMSE (↓)	PCC (↑)	RMSE (↓)	PCC (↑)	RMSE (↓)	PCC (↑)	RMSE (↓)	PCC (↑)
RF	1.212 ± 0.017	0.905 ± 0.003	1.347 ± 0.058	0.881 ± 0.010	2.671 ± 0.579	0.406 ± 0.256	2.906 ± 0.220	0.370 ± 0.138
SVM	1.126 ± 0.016	0.918 ± 0.002	1.346 ± 0.062	0.881 ± 0.011	2.268 ± 0.437	0.520 ± 0.177	2.685 ± 0.350	0.450 ± 0.095
GraphDRP (Nguyen *et al.* 2022)	1.217 ± 0.014	0.904 ± 0.002	1.457 ± 0.050	0.859 ± 0.010	2.354 ± 0.394	0.466 ± 0.163	2.844 ± 0.458	0.356 ± 0.108
PathDNN (Deng *et al.* 2020)	1.154 ± 0.011	0.928 ± 0.002	1.595 ± 0.076	0.862 ± 0.014	3.257 ± 0.666	0.336 ± 0.271	3.065 ± 0.471	0.383 ± 0.128
Precily (Chawla *et al.* 2022)	1.138 ± 0.016	0.917 ± 0.002	1.471 ± 0.063	0.856 ± 0.013	2.825 ± 0.400	0.362 ± 0.109	2.765 ± 0.344	0.426 ± 0.093
DRPreter (Shin *et al.* 2022)	1.104 ± 0.078	0.922 ± 0.011	1.495 ± 0.070	0.852 ± 0.013	2.473 ± 0.360	0.443 ± 0.175	2.745 ± 0.393	0.411 ± 0.148
DeepCoVDR (Huang *et al.* 2023)	1.019 ± 0.015	0.935 ± 0.002	1.394 ± 0.069	0.875 ± 0.012	2.754 ± 0.245	0.387 ± 0.200	3.001 ± 0.423	0.350 ± 0.139
DeepTTA (Jiang *et al.* 2022)	0.974 ± 0.010	0.940 ± 0.001	1.352 ± 0.060	0.881 ± 0.011	2.322 ± 0.496	0.502 ± 0.198	2.806 ± 0.512	0.404 ± 0.125
CSG^2^A (Ours)	**0.942 ± 0.011**	**0.944 ± 0.001**	**1.342 ± 0.059**	**0.883 ± 0.010**	**2.119 ± 0.397**	**0.611 ± 0.140**	**2.442 ± 0.304**	**0.577 ± 0.082**

Model performances are assessed using RMSE and PCC metrics. The reported values represent averages and standard deviations across 10 cross-validation. The best performance is highlighted in bold, and the second-best performance is underlined. (RMSE, root mean square error; PCC, Pearson correlation coefficient).

Additional experiments on the NCI-60 dataset (Shoemaker 2006) with cell level 50% growth inhibitory concentration (GI50) prediction also demonstrated the outperformance of the CSG^2^A network compared to baseline models (Supplementary Table S2). These results underscore the adaptability of our model in diverse application contexts, especially in the challenging grounds of drug discovery.

### 4.2 Pretraining gene–gene attention from transcriptomic landscape improves prediction

**Figure 2.**
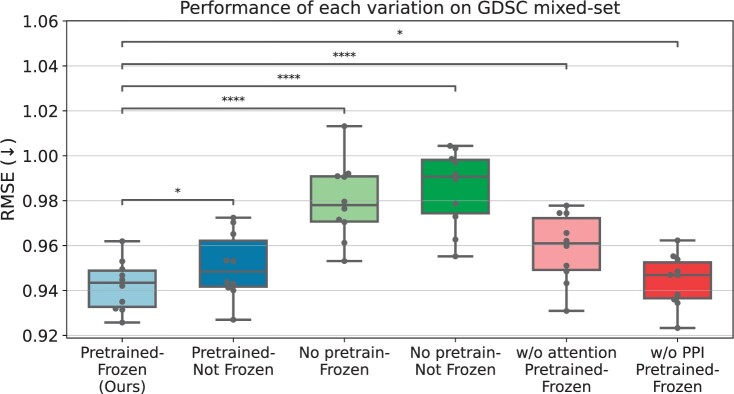
Performances of CSG^2^A network with variations in each modules. Our attention-based approach, equipped with LINCS L1000 pretraining, PPI network information and frozen parameters, shows statistically significant performance enhancements in GDSC dataset mixed-set (*paired *t*-test *P*-value < .05, ****paired *t*-test *P*-value < 1e−4).

Our additional experiments reveal the significant impact of transfer learning from LINCS using our CSG^2^A network on enhancing drug response prediction performances. Figure 2 illustrates the model performances of our model on GDSC mixed-set in RMSE, based on the variations on the inclusion of pretraining and freezing.

An intriguing finding is that our model achieved its best performance when pretrained on LINCS, and the model parameters were frozen during fine-tuning. Importantly, our analysis exposes a statistically significant drop in performance (paired *t*-test *P*-value < .05) on 10CV rounds when layers were fine-tuned (mean RMSE 0.951) compared to the frozen CSG^2^A network (mean RMSE 0.942). This is contrary to the conventional trend in transfer learning for deep models, where fine-tuning the parameters typically improves performance. This suggests that the gene–gene interactions learned from the transcriptomic landscape hold substantial value, and losing this context during fine-tuning does not contribute to performance improvement.

Moreover, pretrained models consistently exhibited superior performances compared to non-pretrained models, surpassing even the fine-tuned unfrozen model (mean RMSE 0.985). This indicates the importance of LINCS pretraining in capturing the dynamics of gene–gene network perturbation, emphasizing its role in boosting predictive capabilities. We also observed a statistically significant decrease in performance when the PPI network information was not integrated into the attention score matrix (mean RMSE 0.945), emphasizing the importance of informative prior knowledge.

Lastly, the performance comparison between the CSG^2^A network and a plain linear prediction model, which does not perform attention but just adds the chemical condition representation and the basal gene expression, empirically demonstrates the importance of utilizing the attention module for best performance. Additionally, we have performed performance comparison of prediction for LINCS L1000 dataset prediction, and also observed that our attention-based approach shows superior performance compared to the plain linear model, shown in Supplementary Table S3.

In addition, we conducted a zero-shot investigation using the LINCS-pretrained CSG^2^A network on the GDSC dataset. By evaluating the Euclidean distance between gene expressions before and after treatment, we observed that the perturbed gene expression points of the sensitive group were significantly farther from the basal gene expression points compared to the resistant group, demonstrating the capability of our LINCS-pretrained model to be capable of understanding the perturbation in cellular conditions at the gene level. Further experimental details and results are provided in Supplementary Methods and Supplementary Fig. S1.

Overall, these observations underscore the success of our trans-level transfer learning strategy with attention module, highlighting the effective integration of knowledge from transcriptomic landscape to enhance predictions for cell line-level drug responses.

### 4.3 Condition-specific gene attention aligns with MoA of drugs

**Figure 3.**
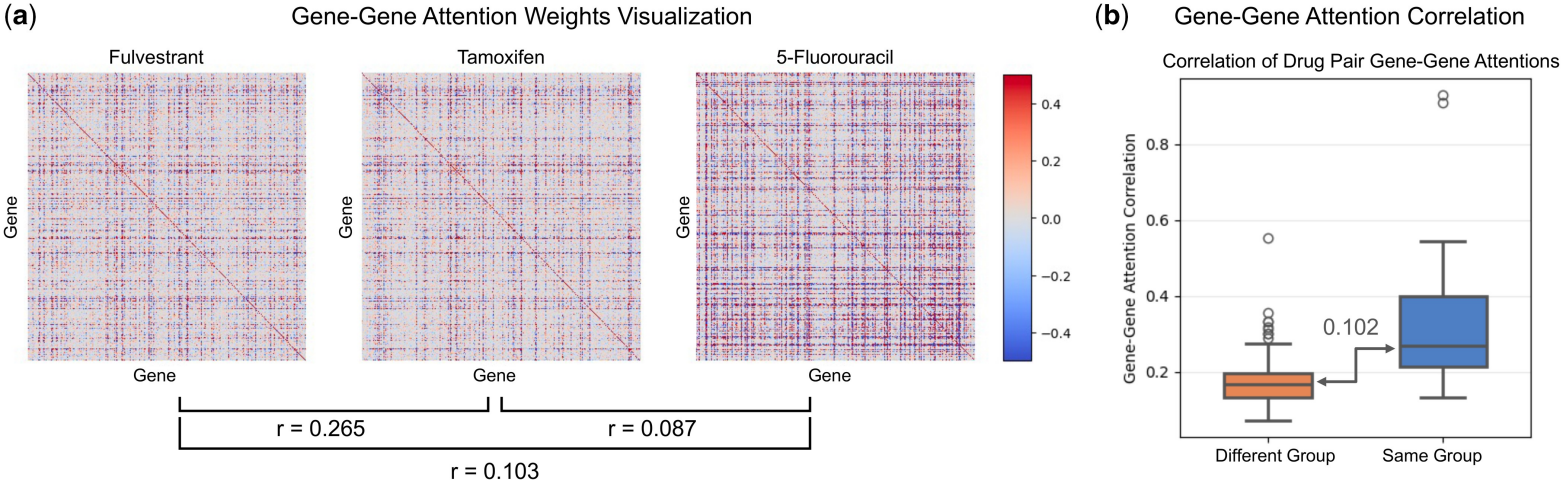
Analysis of gene–gene attention scores. (a) Visualization of gene–gene attention values for three drugs: fulvestrant, tamoxifen, and 5-fluorouracil. The pairwise correlation of the attention matrix is presented below. (b) Correlation of gene–gene attentions among drugs within the same group and across different groups. The correlation within drug pairs from the same group is significantly higher than those from different groups (*r*: Pearson correlation coefficient).

In this section, we showcase a case study that underscores the effectiveness of our model in capturing perturbed gene–gene interactions aligned with the mechanism of action (MoA) of drugs. The gene–gene attention weights for various drugs are visualized in Fig. 3a.

We conducted the analysis by collecting data treated with either fulvestrant or 5-fluorouracil (5-FU) from the test set, resulting in choosing 151 and 99 samples, respectively. Subsequently, we generated the corresponding numbers of attention maps for 151 and 99 samples as $g_{0}$s for the two drugs from the GDSC fine-tuned model. These attention maps were then averaged for each drug to capture a common signal among the diverse basal gene expressions.

Following this step, we extracted the top 1000 gene pairs with the highest pairwise attention score matrix and all the genes within the pairs were utilized for downstream enrichment analysis. The extracted most-perturbed gene sets were then passed down to gene set enrichment analysis for identifying the pathways predominantly influenced by chemical treatment, using the Enrichr (Xie *et al.* 2021) with Wikipathways (Agrawal *et al.* 2024) gene sets. The detailed analysis procedure is described in Supplementary Methods section.

In the case of Fulvestrant, an FDA-approved selective estrogen receptor degrader (SERD), our model highlighted pathways such as integrated breast cancer pathway (WP1984) and apoptosis (WP254) (Alves *et al.* 2016). These findings intricately align with Fulvestrant’s MoA, targeting estrogen receptor signaling and exhibiting anti-cancer effects, particularly in breast cancer (Carlson 2005). Similarly, 5-FU, recognized as an anti-metabolite regulating nucleotide synthesis essential for DNA replication (Longley *et al.* 2003), manifested perturbations in pathways such as DNA damage response (WP707) (De Angelis *et al.* 2006) and apoptosis modulation by HSP70 (WP384) (Grivicich *et al.* 2007). The full list of enriched pathways is listed in Supplementary Table S4. In addition, the identical enrichment analysis on the PPI network-removed CSG^2^A network yielded pathways that were not aligned with the MoA of fulvestrant and 5-FU (Supplementary Table S5), demonstrating on how the PPI network guides the attention map with biological prior knowledge. Moreover, performing the same investigation with only the “potent” samples, defined as samples with the lowest 25% IC50 values, resulted in enriched pathways that were more relevant to the MoA of the two drugs, with smaller scales of adjusted *P*-values (Supplementary Table S4).

To provide a more systematic demonstration, we explored the similarity in gene–gene attention maps concerning the MoA of drugs. Categorizing anti-cancer drugs into groups, such as DNA cleaving drugs, cross-linking drugs, intercalating drugs, topoisomerase inhibitors, antimetabolites, antitubulin drugs, and tyrosine kinase inhibitors, we observed that drugs sharing MoA exhibited significantly higher correlation in gene–gene attention patterns compared to drugs with differing MoAs (Fig. 3b). The average correlation between drugs in different categories was 0.169, while drug pairs within the same category showed an average correlation of 0.338 resulting in median difference of 0.102. This tendency is also evident in Fig. 3a, where the attention weights between fulvestrant and tamoxifen, both hormone therapy agents, are akin than with 5-FU, an anti-metabolite agent, as shown by the Pearson correlation coefficient. Once again, when we conducted the identical investigation using the PPI network-removed CSG^2^A network, we observed a notable change in the systemic correlation difference, with the difference in median correlations between samples within the same group and those within different groups decreasing from 0.102 to 0.064 (Supplementary Fig. S2). This outcome suggests that the removal of the PPI network knowledge resulted in a decline in the model’s ability to effectively distinguish the MoA of the drugs.

Overall, these comprehensive analyses reinforce the model’s ability to discern condition-specific gene interactions aligned with the diverse MoAs of anti-cancer drugs.

### 4.4 Predicted gene expressions by CSG^2^A align with the MoA of treated compounds

To further validate the effectiveness of our CSG^2^A network in translating knowledge from the transcriptome level to the cell level, we conducted a comprehensive analysis on the predicted chemical-induced gene expression profiles. To achieve this, we employed two key analyses: differentially expressed gene (DEG) analysis and gene set enrichment analysis (GSEA).

Starting with the test set predictions from the GDSC dataset, we utilized our model to predict gene expressions based on the basal gene expression profile and input compound. Subsequently, we categorized the dataset into two groups based on IC50 values: high-IC50 and low-IC50. We then performed DEG analysis on the resulting gene expression profiles. Focusing on two most-frequently occurring drugs in the test set, oxaliplatin and fulvestrant, we conducted a gene set enrichment analysis using the Enrichr (Xie *et al.* 2021) with Wikipathways (Agrawal *et al.* 2024) gene sets. The top five enriched pathways for the “sensitive” low-IC50 group, based on adjusted *P*-values, are summarized in Supplementary Table S6.

Oxaliplatin, a platinum-based chemotherapeutic drug, forms DNA adducts and induces DNA damage for its anti-cancer effects (Alcindor and Beauger 2011). The enriched over-expressed pathways from our model’s predicted transcriptomic profiles include DNA Mismatch Repair (WP531), DNA Replication (WP466), Nucleotide Excision Repair (WP4753), and G1 to S cell cycle control (WP45), directly align with the known MoA of oxaliplatin. Additionally, the down-regulation of pathways of Chromosomal and microsatellite instability in colorectal cancer (WP4216) and Pancreatic adenocarcinoma pathway (WP4263) suggests its potential therapeutic efficacy in these cancers, consistent with the drug’s clinical indications (Comella *et al.* 2009; Conroy *et al.* 2023). The results highlight the capability of CSG^2^A in capturing and interpreting complex cellular responses at both genomic and functional pathway levels.

A similar pattern emerges in the case of fulvestrant, a selective estrogen receptor degrader (SERD). First approved by the FDA in 2002, fulvestrant’s MoA is reflected in the most enriched pathway for the predicted suppressed genes in the low-IC50 group: Estrogen signaling pathway (WP712). The down-regulation of the Endometrial cancer pathway, associated with estrogen receptor regulation, further emphasizes the drug’s impact on estrogen-related mechanisms. The observed over-expression of growth factor pathways including PDFG (WP2526) and VEGF (WP3888), coupled with the down-regulation of the EGFR inhibitor resistance pathway (WP4806), suggests cellular adaptations in the absence of estrogen-mediated growth signals. The well-established understanding of the intricate cross-talk between the estrogen signaling pathway and the PDGF, VEGF, and EGFR pathways (Skandalis *et al.* 2014; Lee *et al.* 2001) provides insights into these observations.

In summary, our analysis of oxaliplatin and fulvestrant showcases that the predicted gene expressions by CSG^2^A consistently align with the known MoA of these drugs, demonstrating the model’s capability to capture and interpret complex cellular responses across both cell line and transcriptomic levels.

### 4.5 Translation to patient-level transcriptome data in TCGA

In order to assess the model’s ability to translate its knowledge gained from the GDSC dataset to predict drug responses in the context of patient-specific data from TCGA, we leveraged the GDSC-fine-tuned CSG^2^A model to predict IC50 values for various anti-cancer drugs given patients’ basal tumor tissue gene expression profiles (Supplementary Methods). The assessment relies on the well-established notion that lower IC50 values indicate drug sensitivity, with responders exhibiting a lower IC50 distribution than non-responders.

As part of the performance assessment, one-sided *t*-test was employed to compare the lower distribution of predicted IC50 values between responders and non-responders. The model’s ability to distinguish responders from non-responders was further evaluated through a comparative analysis with DeepTTA and GraphDRP, also trained on the GDSC dataset.

**Figure 4.**
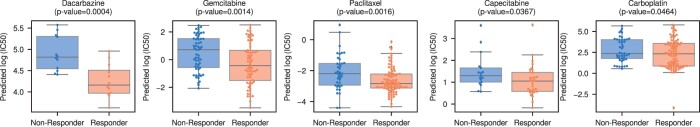
Patient response predictions in TCGA dataset. Using the GDSC-fine-tuned CSG^2^A Network, we predicted drug responses based on patient transcriptomic data from the TCGA dataset. The model successfully differentiated the lower distribution of IC50 values in responders compared to non-responders for dacarbazine, gemcitabine, paclitaxel, capecitabine, and carboplatin as determined by a one-sided *t*-test (*P*-value < .05).

Among 13 drugs with over 10 responders and non-responders each, our model demonstrated enhanced discriminative power (Fig. 4 and Supplementary Table S7). Specifically, it distinguished responses for five drugs with *p*-values below .05 and seven drugs below .1. In contrast, DeepTTA and GraphDRP achieved fewer significant differentiations, with two and three drugs below p-values of 0.05, and three and four drugs below *p*-values of 0.1, respectively. These results underscore the efficacy of our model in transferring knowledge for predicting patient responses and its outperformance in comparison to existing methods.

## 5 Conclusion

In conclusion, our study showcases the efficacy of adeptly capturing intricate condition-specific gene interactions with attention mechanism. This novel CSG^2^A learns from both data and network priors, and uniquely leverages the condition-specific attention scores directly as the neural network parameters.

The success of our trans-level transfer learning strategy is demonstrated in the enhanced drug response predictions, seamlessly integrating knowledge acquired from the transcriptomic landscape of LINCS L1000 dataset. Our pretrained models consistently outperform non-pretrained counterparts, emphasizing the pivotal role of LINCS pretraining. An intriguing observation highlights the value of freezing model parameters during fine-tuning, preserving the context of gene interactions and contributing to optimal performance. Moreover, case studies on gene–gene attention scores and DEG analyses validate the interpretability and biological relevance of our model, aligning well with known drug mechanisms.

Potential future directions may include exploring the extension of our approach to broader patient populations, consideration of multi-omics data, and the integration of external knowledge bases for an even more comprehensive understanding of drug response mechanisms.

As we conclude, we believe our model demonstrates adaptability to diverse application contexts, laying the groundwork for future investigations of the transfer learning framework into more complex biological scenarios.

## Supplementary Material

btae249_Supplementary_Data

## Data Availability

All datasets utilized in this study are publicly available, and the source code is accessible online at https://github.com/eugenebang/CSG2A.
