# Supplementary material for: Transfer learning of condition-specific perturbation in gene interactions improves drug response prediction
Source: Bioinformatics. 2024 Jun 28;40(Suppl 1):i130–9. doi: 10.1093/bioinformatics/btae249 (PMC11256952; doi:10.1093/bioinformatics/btae249)
Supplement: btae249_Supplementary_Data [file btae249_supplementary_data.pdf]

SUPPLEMENTARY INFORMATION FOR: Transfer Learning of Condition-Specific Perturbation in Gene Interactions Improves Drug Response Prediction

Dongmin Bang[1,2,†], **Bonil Koo**[1,2,†], **and Sun Kim**[1,2,3,4,*]

[1]Interdisciplinary Program in Bioinformatics, Seoul National University, Seoul, Republic of Korea
[2]AIGENDRUG Co., Ltd., Seoul, Republic of Korea
[3]Department of Computer Science and Engineering, Seoul National University, Seoul, Republic of Korea
[4]Interdisciplinary Program in Artificial Intelligence, Seoul National University, Seoul, Republic of Korea
[†]These authors contributed equally to this work
[*]For whom the correspondence should be: sunkim.bioinfo@snu.ac.kr

Contents

1 Supplementary Tables **2**
1.1 Supplementary Table S1. Hyperparameter search space . . . 2
1.2 Supplementary Table S2. Additional validation on NCI-60 dataset . . . 3
1.3 Supplementary Table S3. Prediction performance on LINCS pretraining . . . 4
1.4 Supplementary Table S4. Attention analysis results . . . 5
1.5 Supplementary Table S5. Attention analysis results with PPI-removed model . . . 6
1.6 Supplementary Table S6. Predicted gene expression enrichment results . . . 7
1.7 Supplementary Table S7. Distinguishing drug responders from non-responders in TCGA dataset . . . 8
1.8 Supplementary Table S8. Performance of Ablation Models in GDSC Dataset . . . 9

2 Supplementary Figures **10**
2.1 Supplementary Fig. S1. Zero-shot prediction of response levels (IC50) from inferred perturbed gene expression . . . 10
2.2 Supplementary Fig. S2. Attention map correlation analysis with variance in PPI network utilization . . . 11

3 Supplementary Methods **12**
3.1 Supplementary Methods 1. Cell-level drug response prediction models . . . 12
AutoEncoder-based Models • Graph Neural-Network Based Models • Other Approaches
3.2 Supplementary Methods 2. Details on the four data partitioning schemes . . . 12
3.3 Supplementary Methods 3. Gene set enrichment analysis on condition-specific gene attention scores . . . 12
3.4 Supplementary Methods 4. Prediction of patient drug responsiveness in TCGA data . . . 13

References **13**

1 Supplementary Tables

1.1 Supplementary Table S1. Hyperparameter search space

Parameter	Search space	Selected value
Learning rate	[1e-5, 1e-4, 1e-3]	low = 0.003, high = 0.03
Learning rate scheduler	{'constant', 'cosine anneal'}	'cosine anneal'
Early stopping patience	{5, 10, 20}	20
Batch size (LINCS L1000 Dataset)	{32, 64, 80}	80
Batch size (GDSC Dataset)	{32, 64, 128, 256}	128
Self-Attention Weight Dimension (d_q)	{4, 16, 64}	64
Projection Layer Hidden Dimension	{256, 512, 1024}	512
IC50 Prediction MLP Hidden Layer Count	{0, 1, 2}	2
IC50 Prediction MLP Hidden Layer Dimension	{32, 64, 128}	64

1.2 Supplementary Table S2. Additional validation on NCI-60 dataset

Supplementary Table S2. Additional validation on NCI-60 dataset[1] retrieved from the supplementary data by Chen et al[2]. (https://github.com/Jinyu2019/Suppl-data-BBpaper). Unlike the GDSC dataset, the processed NCI-60 provides drug sensitivity data in the form of the 50% growth inhibitory concentration (GI50). We evaluated the performance of our method alongside existing drug response prediction methods, and our method showed superior performance. The best performance is highlighted in bold, and the second-best performance is underlined. (RMSE: Root Mean Square Error; PCC: Pearson Correlation Coefficient)

Models	RMSE (↓)	PCC (↑)
RF	0.833 ± 0.032	0.403 ± 0.067
SVM	0.835 ± 0.034	0.403 ± 0.073
DRPreter	0.860 ± 0.029	0.327 ± 0.123
GraphDRP	0.838 ± 0.021	0.390 ± 0.051
DeepCoVDR	0.812 ± 0.021	0.482 ± 0.044
DeepTTA	<u>0.773 ± 0.028</u>	<u>0.548 ± 0.043</u>
CSG^{2}A	**0.759 ± 0.022**	**0.564 ± 0.031**

1.3 Supplementary Table S3. Prediction performance on LINCS pretraining

Supplemenatry Table S3. Prediction performance during LINCS pretraining is evaluated using two models: a plain linear neural network and the $\texttt{CSG}^2\texttt{A}$ Network, which incorporates attention weights as neural network parameters. The comparison is conducted using two metrics, Mean Squared Error (MSE) and Pearson Correlation Coefficient (PCC).

**Models**	MSE (↓)	PCC (↑)
Pain Linear Neural Network	1.2967 ± 0.01115	0.2685 ± 0.00117
CSG^2A Network	**1.1861 ± 0.00799**	**0.3776 ± 0.00328**

1.4 Supplementary Table S4. Attention analysis results

Supplementary Table S4. The top 10 enriched pathways on highly perturbed gene sets for fulvestrant and 5-fluorouracil, ranked by adjusted p-value. The table at the top presents the enrichment results using the whole GDSC testset samples, while the table below depicts the results using only the 'potent' samples, defined as samples with the lowest 25% IC50 values. Both sets of results demonstrate the alignment of the enriched pathways with the Mode of Action of the two drugs. We can also observe that the 'potent' set shows greater relevance with lower scales of adjusted p-values, indicating higher confidence towards the enrichment results. (adj. p.: adjusted p-value)

Whole GDSC test samples					
Fulvestrant (151 samples)			5-Fluorouracil (99 samples)		
Rank	Attention-enriched pathway	adj. p.	Rank	Attention-enriched pathway	adj. p.
1	Integrated Cancer Pathway WP1971	2.3e-5	1	DNA damage response WP707	1.5e-3
2	Integrated breast cancer pathway WP1984	2.3e-5	2	Gastrin signaling pathway WP4659	1.5e-3
3	DNA damage response WP707	2.7e-4	3	miRNA regulation of DNA damage response WP1530	1.5e-3
4	ErbB signaling pathway WP673	2.7e-4	4	Apoptosis-related network due to altered Notch3 in ovarian cancer WP2864	2.2e-3
5	RAC1/PAK1/p38/MMP2 Pathway WP3303	2.7e-4	5	Alzheimer's disease WP2059	2.5e-3
6	miRNA regulation of DNA damage response WP1530	3.0e-4	6	Apoptosis Modulation by HSP70 WP384	2.8e-3
7	Apoptosis WP254	9.0e-4	7	Unfolded protein response WP4925	5.5e-3
8	Endometrial cancer WP4155	9.0e-4	8	miRNA regulation of p53 pathway in prostate cancer WP3982	5.5e-3
9	Retinoblastoma gene in cancer WP2446	9.6e-4	9	Apoptosis WP254	1.3e-2
10	Pancreatic adenocarcinoma pathway WP4263	1.5e-3	10	Retinoblastoma gene in cancer WP2446	1.4e-2

'Potent' GDSC test samples					
Fulvestrant (37 samples)			5-Fluorouracil (24 samples)		
Rank	Attention-enriched pathway	adj. p.	Rank	Attention-enriched pathway	adj. p.
1	Integrated Cancer Pathway WP1971	9.0e-6	1	DNA damage response WP707	2.8e-4
2	Integrated breast cancer pathway WP1984	9.0e-6	2	miRNA regulation of DNA damage response WP1530	2.8e-4
3	DNA damage response WP707	5.0e-5	3	Gastrin signaling pathway WP4659	8.3e-4
4	EGF/EGFR signaling pathway WP437	5.0e-5	4	Retinoblastoma gene in cancer WP2446	6.0e-3
5	ErbB signaling pathway WP673	5.0e-5	5	Unfolded protein response WP4925	9.0e-3
6	RAC1/PAK1/p38/MMP2 Pathway WP3303	5.0e-5	6	miRNA regulation of p53 pathway in prostate cancer WP3982	9.0e-3
7	Retinoblastoma gene in cancer WP2446	5.0e-5	7	Apoptosis-related network due to altered Notch3 in ovarian cancer WP2864	1.8e-3
8	miRNA regulation of DNA damage response WP1530	5.2e-5	8	G1 to S cell cycle control WP45	2.7e-3
9	Apoptosis WP254	1.6e-4	9	LncRNA involvement in canonical Wnt signaling and colorectal cancer WP4258	2.7e-2
10	Endometrial cancer WP4155	1.7e-4	10	MAP3K1 role in promoting and blocking gonadal determination WP4872	2.7e-2

1.5 Supplementary Table S5. Attention analysis results with PPI-removed model

Supplementary Table S5. Top-10 Enriched pathways on highly perturbed gene sets for fulvestrant and 5-fluorouracil from the PPI-removed model, ranked by adjusted p-value. For the training of the PPI-removed model, the PPI adjacency matrix was not added to the attention score matrix of the $\texttt{CSG}^2\texttt{A}$ network during both the pretraining on LINCS L1000 and finetuning on GDSC datasets. (adj. p.: adjusted p-value)

Fulvestrant			5-Fluorouracil		
Rank	Attention-enriched pathway	adj. p.	Rank	Attention-enriched pathway	adj. p.
1	Gastrin signaling pathway WP4659	2.1e-4	1	Acute viral myocarditis WP4298	3.8e-3
2	Amplification and expansion of oncogenic pathways as metastatic traits WP3678	1.4e-3	2	Head and Neck Squamous Cell Carcinoma WP4674	8.0e-3
3	MFAP5 effect on permeability and motility of endothelial cells via cytoskeleton rearrangement WP4560	4.2e-3	3	Thymic Stromal LymphoPoietin (TSLP) Signaling	1.1e-2
4	Netrin-UNC5B signaling pathway WP4747	1.5e-2	4	mRNA Processing WP411	1.1e-2
5	Pathogenic Escherichia coli infection WP2272	1.9e-2	5	NRF2-ARE regulation WP4357	1.9e-2
6	Cardiac Progenitor Differentiation WP2406	2.2e-2	6	Pre-implantation embryo WP3527	1.9e-2
7	Cell cycle WP179	3.4e-2	7	Nucleotide metabolism WP404	2.3e-3
8	Pathways affected in adenoid cystic carcinoma WP3651	3.5e-2	8	Pregnane X receptor pathway WP2876	2.3e-2
9	Follicle Stimulating Hormone (FSH) signaling pathway WP2035	3.5e-2	9	Angiogenesis WP1539	2.9e-2
10	Gastric Cancer Network 1 WP2361	3.5e-2	10	Aryl Hydrocarbon Receptor Pathway WP2873	2.9e-2

1.6 Supplementary Table S6. Predicted gene expression enrichment results

Supplementary Table S6. Top-5 Enriched pathways on over-expressed and suppressed gene sets for oxaliplatin and fulvestrant, ranked by adjusted p-value. (adj. p.: adjusted p-value)

		Oxaliplatin			
Rank	Over expressed gene-enriched pathway	adj. p.	Rank	Suppressed gene-enriched pathway	adj. p.
1	DNA Mismatch Repair WP531	4.5e-5	1	Chromosomal and microsatellite instability	1.2e-3
2	Retinoblastoma gene in cancer WP2446	9.0e-5	2	Pancreatic adenocarcinoma pathway WP4263	2.2e-3
3	DNA Replication WP466	3.3e-4	3	Apoptosis-related network due to altered Notch3 in ovarian cancer WP2864	6.6e-3
4	Nucleotide Excision Repair WP4753	3.3e-4	4	EGF/EGFR signaling pathway WP437	6.6e-3
5	G1 to S cell cycle control WP45	1.7e-3	5	Gastrin signaling pathway WP4659	6.6e-3

		Fulvestrant			
Rank	Over expressed gene-enriched pathway	adj. p.	Rank	Suppressed gene-enriched pathway	adj. p.
1	PDGF Pathway WP2526	3.1e-3	1	DNA damage response (only ATM dependent) WP710	3.5e-3
2	Mammary gland development pathway - Puberty (Stage 2 of 4) WP2814	8.9e-3	2	Estrogen signaling pathway WP712	3.5e-3
3	Photodynamic therapy-induced HIF-1 survival signaling WP3614	8.9e-3	3	Hepatitis B infection WP4666	3.5e-3
4	RAC1/PAK1/p38/MMP2 Pathway WP3303	8.9e-3	4	Endometrial cancer WP4155	1.2e-2
5	VEGFA-VEGFR2 Signaling Pathway WP3888	8.9e-3	5	EGFR Tyrosine Kinase Inhibitor Resistance WP4806	1.9e-2

1.7 Supplementary Table S7. Distinguishing drug responders from non-responders in TCGA dataset

Supplementary Table S7. The table presents the results of evaluating the GDSC-finetuned CSG^{2}A model's ability to predict drug responses in patient data from TCGA. Our model outperformed DeepTTA and GraphDRP in distinguishing responders from non-responders, demonstrating enhanced discriminative power with significant p-values for five drugs below 0.05 and seven drugs below 0.1. These findings underscore the efficacy of our model in transferring knowledge for predicting patient responses.

Model	Drugs with p <0.05		Drugs with p <0.1	
	Drugs	Count	Drugs	Count
CSG^{2}A	Dacarbazine, Gemcitabine, Paclitaxel, Capecitabine, Carboplatin	5 (38.5%)	Dacarbazine, Gemcitabine, Paclitaxel, Capecitabine, Carboplatin, Docetaxel, Leucovorin	7 (53.8%)
GraphDRP	Gemcitabine, Docetaxel, Cisplatin	3 (23.1%)	Gemcitabine, Docetaxel, Cisplatin, Leucovorin	4 (30.8%)
DeepTTA	Cisplatin, Dacarbazine	2 (15.4%)	Cisplatin, Dacarbazine, Docetaxel	3 (23.1%)

1.8 Supplementary Table S8. Performance of Ablation Models in GDSC Dataset

Supplementary Table S8. Performance in ablation studies. Results on removal of linear scaling and application of batch correction. Transfer learning with linear scaling layer removed shows decrease in performances, especially in the difficult split settings (cell line-blind, drug-blind, and disjoint-set). Additionally, we have conducted a batch correction (Combat[3]) applied experiment with LINCS and GDSC, compared the results and confirmed that there is no loss of prediction power when using a linear scaling layer compared to the batch correction applied data. Furthermore, when we trained and evaluated the model without considering dosage and time information, we observed a decrease in performance.

Models	Mixed-set	Cell line-blind	Drug-blind	Disjoint-set
	RMSE (↓)	RMSE (↓)	RMSE (↓)	RMSE (↓)
CSG^{2}A (proposed)	0.942 ± 0.011	1.349 ± 0.063	2.198 ± 0.470	2.442 ± 0.304
- Batch correction instead of linear scaling	0.943 ± 0.011	1.351 ± 0.054	2.206 ± 0.446	2.443 ± 0.306
- Linear scaling layer removed	0.944 ± 0.013	1.353 ± 0.061	2.296 ± 0.475	2.546 ± 0.296
- Dose and time information removed	0.948 ± 0.013	1.347 ± 0.062	2.278 ± 0.441	2.560 ± 0.264

2 Supplementary Figures

2.1 Supplementary Fig. S1. Zero-shot prediction of response levels (IC50) from inferred perturbed gene expression

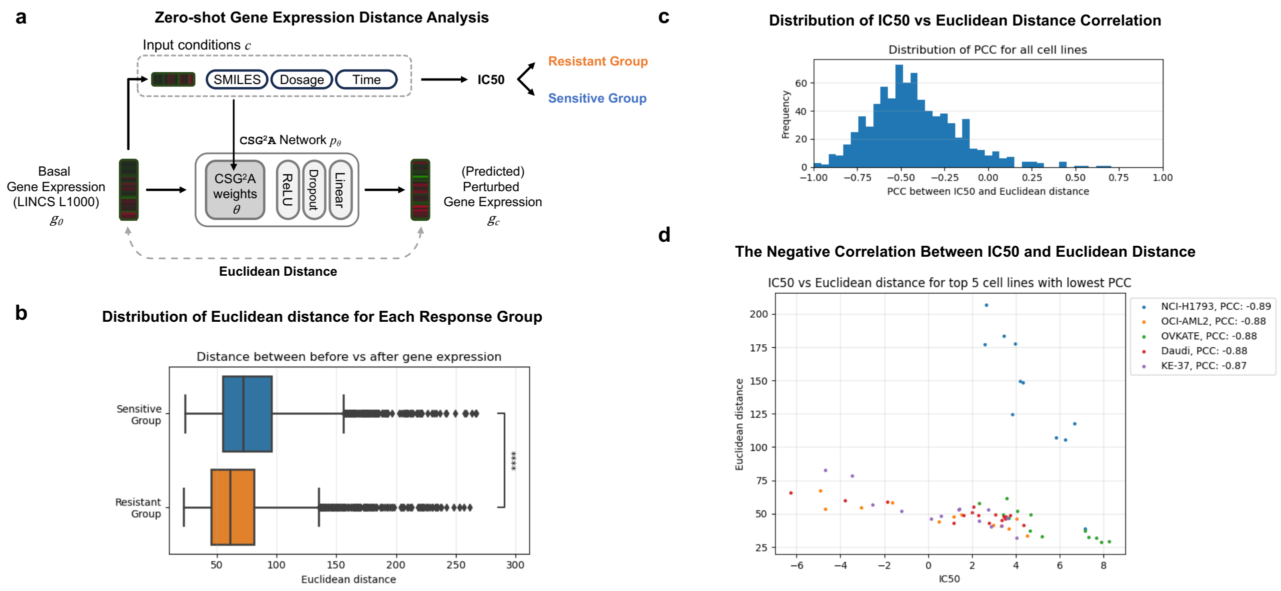

Supplementary Fig. S1. Zero-shot prediction of drug response from perturbed gene expression. (**a**) The zero-shot prediction framework based on inferred perturbed gene expression using the LINCS-pretrained $\texttt{CSG}^2\texttt{A}$ network. (**b**) Distribution of Euclidean distance for each response group. Statistical tests have verified that the resistant group exhibited significantly closer proximity to the basal gene expression, as evidenced by a one-sided t-test yielding a p-value of 5.1E-97. (**c**) Distribution of IC50 vs Euclidean distance correlations. The distribution of Among the 803 cell lines, 756 cell lines (94.1%) showed negative correlations between the Euclidean distance and the logIC50 value of the samples from the corresponding cell line. (**d**) The negative correlation between IC50 and Euclidean distance. The results suggest that lower the IC50 value, indicating higher sensitivity to the input compound, the greater the distance observed in Euclidean gene expression space created the LINCS-pretrained $\texttt{CSG}^2\texttt{A}$ network. (PCC: Pearson Correlation Coefficient; ****: t-test p-value $<$ 1E-4)

2.2 Supplementary Fig. S2. Attention map correlation analysis with variance in PPI network utilization

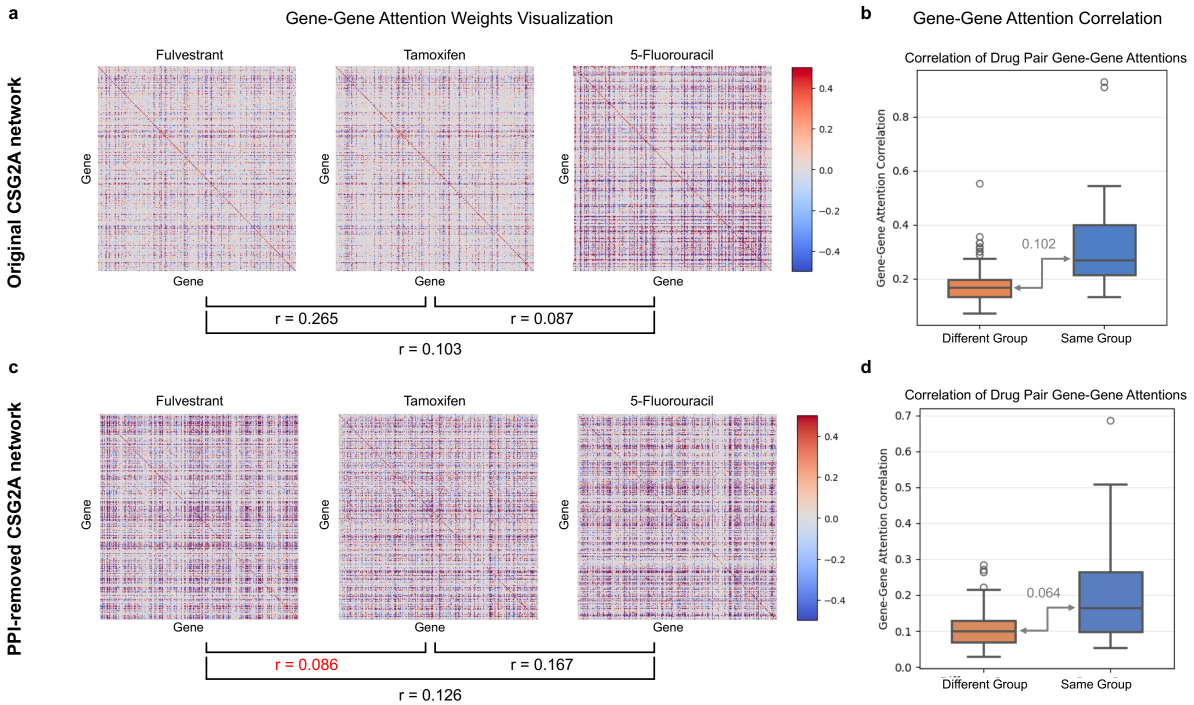

Supplementary Fig. S2. Analysis of Gene-Gene Attention Scores. The upper row displays results obtained from the original $\texttt{CSG}^2\texttt{A}$ network, identical to those presented in Figure 3 of the main text. The lower row depicts results from the PPI-removed variant model. (**a, c**) Visualization of gene-gene attention values for three drugs: fulvestrant, tamoxifen, and 5-fluorouracil. The pairwise correlation of the attention matrix is provided below each visualization. (**b, d**) Correlation of gene-gene attentions among drugs within the same group and across different groups. The correlation within drug pairs from the same group is significantly higher than those from different groups. However, the difference between the 'Same Group' and the 'Different Group' has decreased, with the median difference reducing from 0.102 to 0.064. (r: Pearson Correlation Coefficient)

3 Supplementary Methods

3.1 Supplementary Methods 1. Cell-level drug response prediction models

3.1.1 AutoEncoder-based Models

DeepDR[4] is a AutoEncoder-based deep learning model designed for predicting drug responses in cancer cells based on mutation and expression profiles. The model, incorporating pre-trained encoders and a drug response predictor network, demonstrated its effectiveness in predicting drug responses across various cancer types. However, the model does not use any drug information, making it difficult to use from a new drug development perspective.

Dr.VAE[5] is the first work to attempt the integration of knowledge from transcriptomic level drug response for cell line drug response prediction. Utilizing the Variational AutoEncoder framework, the authors pre-trained models on LINCS L1000 data and applied additional classifiers to predict cell line level drug responses. However, this model lacks consideration for various chemical treatment conditions. Notably, Dr.VAE trains separate models for each drug with fixed dosage and time point, limiting its ability to predict responses for drugs and conditions absent from the LINCS L1000 dataset.

3.1.2 Graph Neural-Network Based Models

GraphDRP[6] introduced a method based on GNNs for drug response prediction by representing drugs as molecular graphs and cell lines as binary vectors of genomic aberrations. It demonstrated the efficacy of graph-based representations in enhancing drug response prediction. However, since 1D convolutional layers are employed to learn cell line representation, interactions between genes cannot be taken into account.

DRPreter[7] is an interpretable model utilizing graph neural networks to predict anticancer drug response. DRPreter incorporates domain knowledge on biological pathways, employs a transformer to detect relationships between pathways and drugs, while also providing insights into the mechanism of action. However, bias can occur when only information about specific pathways and their associated genes is utilized. Also, the fixed structure of pathways limit the model in learning chemical-induced perturbation in gene-gene interaction level.

3.1.3 Other Approaches

PathDNN[8] proposed a pathway-guided deep neural network (DNN) model to predict drug sensitivity in cancer cells. The model, incorporating biological pathway information, demonstrated improved interpretability, highlighting its potential for enhancing understanding and prediction of drug sensitivity in cancer treatment. However, due to the model's reliance on the drug's target information as inputs, it becomes challenging to apply it to drugs lacking such target information.

Precily[9] utilizes a simple DNN that takes pathway scores processed from gene expression profiles and integrates them with drug descriptors, providing insights into the biological mechanisms influencing drug resistance.

DeepTTA[10] integrates transformer-based drug representation learning with a feed-forward network for predicting anti-cancer drug responses using transcriptomic data and drug chemical substructures. However, the model structure mainly focuses on drugs and does not account for interactions between genes.

DeepCoVDR[11] utilizes a graph transformer to encode chemical compounds and feed-forward layers to encode cell-lines. Then, a cross-attention module integrates compound embedding and cell-line embedding by considering their interaction. DeepCoVDR then predicts IC50 value using the representations of the compound, cell-line, and interaction features.

3.2 Supplementary Methods 2. Details on the four data partitioning schemes

Aligning with the comprehensive investigation by Partin et al.[12], we conducted 10-fold validation in four distinct data partitioning schemes to evaluate each models' generalizability to diverse scenarios. The mixed-set scenario (known cancer and drugs) is commonly employed for its simplicity in analyzing and implementing drug response prediction models. The cell line-blind scenario (unknown cell lines and known drugs) is for simulating personalized cancer treatment utility, and the drug-blind scenario (known cancers and unknown drugs) presents challenges in developing novel drugs for cancer treatment. The disjoint-set scenario (unknown cancers and drugs) is used to assess each models' capacity to generalize in the challenging scenarios and its potential application in more clinically relevant settings. The training set comprised 80% of the data, with 10% allocated to the validation set and 10% to the test set.

3.3 Supplementary Methods 3. Gene set enrichment analysis on condition-specific gene attention scores

The proposed Condition-Specific Gene-Gene Attention ($\texttt{CSG}^2\texttt{A}$) module is designed for capturing the gene-gene interactions induced by the basal gene expression and chemical treatment condition. The resulting gene-gene attention scores are directly utilized as neural network parameters for predicting downstream target values, specifically, perturbed gene expression for LINCS L1000 and IC50 values for GDSC.

In this section, we describe the methods to assess the association between known drug mechanisms and the gene-gene attention values. Starting from the test set samples in the GDSC, we extracted the gene-gene attention map of the drugs-of-interest (DOI), fulvestrant and 5-fluorouracil (5-FU). The test set contained a total of 151 fulvestrant-treated samples and 99

5-FU treated samples. The attention maps were aggregated by averaging, resulting in a representative attention map for each drug.

After performing absolute operation on the attention scores, we identified the top 1,000 gene-gene attention values for each drugs as 'most-perturbed gene interactions'. Then all the genes appearing in the 1,000 pairs are selected as 'most-perturbed gene set', prepared for downstream Gene Set Enrichment Analysis (GSEA). Using the widely-used GSEA tool Enrichr[13], we identified enriched pathways using the pathway annotations from WikiPathways[14]. For the two DOIs, top 10 pathways ranked by adjusted p-value are identified, and displayed in Supplementary Table S3.

3.4 Supplementary Methods 4. Prediction of patient drug responsiveness in TCGA data

Using the TCGA[15] classification information of cell lines provided by GDSC[16], transcriptome data corresponding to tumor samples for 21 cancer types were obtained from UCSC Xena[17]. The cancer types include BLCA, BRCA, CESC, COAD, ESCA, GBM, HNSC, KIRC, LGG, LIHC, LUAD, LUSC, MESO, OV, PAAD, PRAD, READ, SKCM, STAD, THCA, and UCEC, with COAD and READ merged into COREAD.

For each cancer type, GDSC and TCGA data were underwent batch correction at the $\log_2$FPKM level using Combat[3]. Then, the values were normalized into robust z-scores using the following equation to be used as input at the same level:

$$z_i = \frac{x_i - \mathrm{median}(X)}{1.4826 \cdot \mathrm{MAD}(X)}, \quad (1)$$

where MAD denotes the median absolute deviation, X represents the expression values for a gene across all samples, x_i is the expression level of a sample i, and z_i is the robust z-score for the gene of sample i. To predict IC50 values for TCGA data, models were trained for each cancer type using GDSC data.

Curated data on drug treatment and responsiveness in TCGA patients were obtained from the supplementary data provided in[18]. Patients were divided into two groups: responder (including complete response and partial response) and non-responder (including stable disease and clinical progressive disease). Within both the responder and the non-responder groups, 13 drugs with more than 10 samples were identified: cisplatin, capecitabine, 5-fluorouracil, oxaliplatin, leucovorin, temozolomide, carboplatin, pemetrexed, gemcitabine, docetaxel, dacarbazine, doxorubicin, and paclitaxel.

The TCGA transcriptome data were inputted into the trained models for each cancer type, inferring IC50 values for each patient and drug pair. Then, one sided t-test was conducted to test the hypothesis that IC50 values in the responder group are lower than those in the non-responder group.

References

1. Shoemaker, R. H. The nci60 human tumour cell line anticancer drug screen. *Nat. Rev. Cancer* **6**, 813–823 (2006).
2. Chen, J. & Zhang, L. A survey and systematic assessment of computational methods for drug response prediction. *Briefings bioinformatics* **22**, 232–246 (2021).
3. Johnson, W. E., Li, C. & Rabinovic, A. Adjusting batch effects in microarray expression data using empirical bayes methods. *Biostatistics* **8**, 118–127 (2007).
4. Chiu, Y.-C. *et al.* Predicting drug response of tumors from integrated genomic profiles by deep neural networks. *BMC medical genomics* **12**, 143–155 (2019).
5. Rampášek, L., Hidru, D., Smirnov, P., Haibe-Kains, B. & Goldenberg, A. Dr. vae: improving drug response prediction via modeling of drug perturbation effects. *Bioinformatics* **35**, 3743–3751 (2019).
6. Nguyen, T., Nguyen, G. T., Nguyen, T. & Le, D.-H. Graph convolutional networks for drug response prediction. *IEEE/ACM transactions on computational biology bioinformatics* **19**, 146–154 (2021).
7. Shin, J., Piao, Y., Bang, D., Kim, S. & Jo, K. Drpreter: Interpretable anticancer drug response prediction using knowledge-guided graph neural networks and transformer. *Int. J. Mol. Sci.* **23**, 13919 (2022).
8. Deng, L. *et al.* Pathway-guided deep neural network toward interpretable and predictive modeling of drug sensitivity. *J. Chem. Inf. Model.* **60**, 4497–4505 (2020).
9. Chawla, S. *et al.* Gene expression based inference of cancer drug sensitivity. *Nat. communications* **13**, 5680 (2022).
10. Jiang, L. *et al.* Deeptta: a transformer-based model for predicting cancer drug response. *Briefings bioinformatics* **23**, bbac100 (2022).
11. Huang, Z., Zhang, P. & Deng, L. Deepcovdr: deep transfer learning with graph transformer and cross-attention for predicting covid-19 drug response. *Bioinformatics* **39**, i475–i483 (2023).

12. Partin, A. *et al.* Deep learning methods for drug response prediction in cancer: predominant and emerging trends. *Front. Medicine* **10**, 1086097 (2023).

13. Xie, Z. *et al.* Gene set knowledge discovery with enrichr. *Curr. protocols* **1**, e90 (2021).

14. Agrawal, A. *et al.* Wikipathways 2024: next generation pathway database. *Nucleic acids research* **52**, D679–D689 (2024).

15. Weinstein, J. N. *et al.* The cancer genome atlas pan-cancer analysis project. *Nat. genetics* **45**, 1113–1120 (2013).

16. Garnett, M. J. *et al.* Systematic identification of genomic markers of drug sensitivity in cancer cells. *Nature* **483**, 570–575 (2012).

17. Goldman, M. J. *et al.* Visualizing and interpreting cancer genomics data via the xena platform. *Nat. biotechnology* **38**, 675–678 (2020).

18. Ding, Z., Zu, S. & Gu, J. Evaluating the molecule-based prediction of clinical drug responses in cancer. *Bioinformatics* **32**, 2891–2895 (2016).
